# Automated extraction and validation of children’s gait parameters with the Kinect

**DOI:** 10.1186/s12938-015-0102-9

**Published:** 2015-12-02

**Authors:** Saeid Motiian, Paola Pergami, Keegan Guffey, Corrie A Mancinelli, Gianfranco Doretto

**Affiliations:** Lane Department of Computer Science and Electrical Engineering, West Virginia University, Morgantown, WV USA; Department of Pediatrics, West Virginia University School of Medicine, Morgantown, WV USA; Department of Biology, West Virginia University, Morgantown, WV USA; Division of Physical Therapy, West Virginia University School of Medicine, Morgantown, WV USA

**Keywords:** Children’s gait analysis, Kinect, GAITRite, Concurrent validity, Dynamic time warping

## Abstract

**Background:**

Gait analysis for therapy regimen prescription and monitoring requires patients to physically access clinics with specialized equipment. The timely availability of such infrastructure at the right frequency is especially important for small children. Besides being very costly, this is a challenge for many children living in rural areas. This is why this work develops a low-cost, portable, and automated approach for in-home gait analysis, based on the Microsoft Kinect.

**Methods:**

A robust and efficient method for extracting gait parameters is introduced, which copes with the high variability of noisy Kinect skeleton tracking data experienced across the population of young children. This is achieved by temporally segmenting the data with an approach based on coupling a probabilistic matching of stride template models, learned offline, with the estimation of their global and local temporal scaling. A preliminary study conducted on healthy children between 2 and 4 years of age is performed to analyze the accuracy, precision, repeatability, and concurrent validity of the proposed method against the GAITRite when measuring several spatial and temporal children’s gait parameters.

**Results:**

The method has excellent accuracy and good precision, with segmenting temporal sequences of body joint locations into stride and step cycles. Also, the spatial and temporal gait parameters, estimated automatically, exhibit good concurrent validity with those provided by the GAITRite, as well as very good repeatability. In particular, on a range of nine gait parameters, the relative and absolute agreements were found to be good and excellent, and the overall agreements were found to be good and moderate.

**Conclusion:**

This work enables and validates the automated use of the Kinect for children’s gait analysis in healthy subjects. In particular, the approach makes a step forward towards developing a low-cost, portable, parent-operated in-home tool for clinicians assisting young children.

## Background

The effectiveness of a rehabilitation regimen can be ensured only if an appropriate monitoring of progress is implemented. This is true even more so for developing children, where detection of gait abnormalities, as well as the adoption of a therapy to correct them, must be validated in a continuous and timely manner to ensure success [1, 2]. Therapy adjustment and gait evaluation in children are further complicated by the natural changes in their motor development, and by their limited ability to provide feedback as precisely as adults, sometimes forcing practitioners to rely on subjective parental information, thus highlighting even further the importance of relying on suitable unbiased assessment tests.

Gait analysis methods [3] are a common way to quantify and assess human locomotion. They have been used successfully as research and clinical tools in many patient populations, including children with cerebral palsy [4], individuals with spinal cord injury [5], or under rehabilitation after stroke [6], and elderly people under risk of falls [7]. Although very useful, gait analysis requires specialized equipment used by expert technicians, typically present in academic research laboratories or large hospitals [8], which poses the problem of timely accessibility of such infrastructure. In addition, costs associated with the set up and administration of gait assessments are reported to be fairly high [9], making it even more difficult to routinely monitor the progress of patients undergoing therapy.

The GAITRite system [10], a walkway with a grid of sensors, is an extensively validated gait analysis tool for both adults [11–14] and children [15–17], which is widely used by practitioners. It provides for the automatic computation of several spatial and temporal gait parameters. Compared to very accurate three-dimensional gait analysis systems (e.g., the Vicon [18]), the GAITRite is easier to operate (especially with children), costs less, has smaller space requirements, and yet is very effective in tracking patient progress. However, it remains a large and expensive device meant to be operated by technicians. This becomes a problem, especially in rural areas, where it is difficult for many families to bring their children into a facility with the appropriate personnel and equipment to detect, monitor and correct gait abnormalities. The availability of an inexpensive, portable, in-home alternative to the GAITRite that is operable by parents would potentially allow clinicians to remotely monitor patient’s progress, and to deliver state-of-the-art low-cost healthcare to an underserved population.

In this work, the Microsoft Kinect [19] is leveraged as a very low-cost sensing device, capable of tracking 20 different body joint locations over time at video rate [20], and it is proposed for children’s gait analysis. To this end, a framework for the automated extraction of gait parameters from Kinect data is developed, and validated on healthy children. Providing accurate and precise measures of gait parameters requires facing the main challenge of designing algorithms that are robust to large amounts of articulated body tracking noise, and that can deal with the variability of tracking data across the population of yang children, and across different age groups. Enabling the implementation of a portable and low-cost system, instead, requires designing computationally efficient algorithms, because of the limited computing power of such platforms.

The proposed framework for estimating gait parameters addresses both of the challenges outlined above. It introduces robust algorithms for the automatic calibration and segmentation of temporal sequences, generated by the 3D locations of body joints. The segmentation accurately decomposes sequences into snippets, corresponding to the strides of the walking child. This is achieved by a probabilistic matching of stride template models, learned offline from training data, coupled with the joint estimation of the global and local temporal scaling of the templates. Computational efficiency, instead, is achieved by augmenting the approach with subsequence matching techniques.

The framework is evaluated in two ways. First, the accuracy and precision in detecting specific temporal instants of the gait cycle are studied. Those include the heel strikes and toe-offs that segment the child’s walk into stride and step cycles. Second, by conducting a study with healthy children, the validity of the gait parameters estimated automatically is established against those computed by the GAITRite, and the repeatability of the approach is also analyzed.

### Related work

Several approaches have been developed for gait analysis outside the clinic [3]. There is a large category of portable approaches based on wearable sensors, such as accelerometers, gyroscopes, pressure sensors, ultrasonic sensors, and others. Some of them can lead to cheaper systems [21], however, they require downloading data to perform the analysis unless additional hardware for wireless data collection is incorporated, and multiple sensors are needed for the analysis of multiple gait parameters. In addition, sensors must be placed correctly and securely, and can be susceptible to noise and interferences due to external factors [3]. Also, it can be very inconvenient for children to wear additional devices, especially those that entail wearing instrumented shoes [22], as further explained below. Currently, the evidence of a simple inexpensive system based on wearable sensors suitable for children’s gait analysis is unclear.

Marker-less vision-based gait analysis approaches are another popular low-cost alternative [23]. They have been studied extensively by the computer vision community for human activity analysis [24] and biometric recognition [25]. Usually, they are based on multiple cameras and can work effectively as fixed in-home installations for the continuous monitoring of gait in elderly patients [26]. However, they require a complex setup with a calibration process and are not adequate to become simple, parent-operated devices.

Other marker-less approaches include those based on time-of-flight cameras, infrared thermography, and pulse-Doppler radars [3, 27]. Those are either too expensive, or not portable and too complex to set up. On the other hand, the Microsoft Kinect (which for Xbox One [28] uses an inexpensive time-of-flight camera, as opposed to those methods referred in [3]), with its software development kit (SDK) makes available a technology for 3D articulated body tracking [20] that is safe, inexpensive, comes in a small package, is straightforward to set up and operate (no need for camera calibration, fix installation or for wearing additional sensors), and is pervasive. Therefore, it offers the opportunity to address the need for a low-cost parent-operated tool for in-home monitoring of gait in children during rehabilitation interventions. This work makes a step forward towards fulfilling such need by introducing and validating a methodology for extracting children’s gait parameters in healthy subjects fully automatically from Kinect tracking data.

The Kinect has been used in several clinical applications related to gait disorders and mobility analysis. It has been used for interventions on the balance ability of injured young male athletes [29], and its reliability and validity for assessing the standing balance was established in [30]. In [31] it was found that for the majority of the considered foot posture index items, the Kinect was more reliable than the traditional visual assessment. More specifically to the functional assessment [32], introduces a methodology to use the Kinect for mapping gait parameters to the Timed-Up-and-Go (TUG) mobility test, and [33] reports a validation and reproducibility study against a standard marker based system for functional assessment activities. Similarly, [34] also considers the TUG test, but they develop a novel algorithm for using the Kinect from the side view, which is particularly suitable for this test, and is capable of locating and tracking up to six joints of a human body. Related to this line of works [35], focusses on establishing the concurrent validity of the Kinect against a 3D motion analysis system for assessing the kinematic strategies of postural control. Compared to the above approaches, ours differs substantially, in that it focusses on developing and validating the extraction of spatiotemporal children’s gait parameters in a fully automated fashion.

More closely related to rehabilitation, the Kinect has been assessed for rehabilitating young adults with motor impairments [36] and with cerebral palsy [37], both in school settings [38]. instead, assessed the concurrent validity of the Kinect for gait retraining using the lateral trunk lean modification model. For patients affected by stroke [39], developed an automated method for measuring the quality of movements in clinically-relevant terms, and [40] examined the reliability of spatiotemporal gait parameters as well as other standard tests, such as the functional reach test, the step test, the 10 m walk test, and the TUG test. For patients with Parkinson’s disease [41], established the accuracy of the Kinect in measuring clinically relevant movements, while [42, 43] developed algorithms aimed at extracting gait parameters to be used for automatically recognizing individuals suspected of having the disease. In patients with multiple sclerosis, [44] showed that ambulation tests using the Kinect are feasible, and can detect clinical gait disturbances. Further references can be found in [45, 46], which review the technical and clinical impact of the Kinect in physical therapy and rehabilitation, with an emphasis on patients with neurological disorders as wel as elderly patients. The studies above do not involve young children, and have very different goals from those of this work.

Kinect-based methods have been used before in clinical applications involving children (e.g., in serious games for rehabilitation [47] and learning [48]), but never for children’s gait analysis. More precisely, Stone and Skubic [49, 50] were the first that advocated the use of Kinect for clinical gait analysis, and applied it for continuous in-home gait monitoring of elderly people. Their approach detected footfalls by analyzing the portion of the foreground depth maps close to the ground plane. The main drawbacks of this approach are the limited number of gait parameters being monitored, as well as a fix installation, requiring the intrinsic and extrinsic calibration of the Kinect. Gabel et al. [51] instead, proposed an easier-to-use approach that also provided a broader set of gait parameters. Those were estimated with a supervised learning method, where an ensemble of regression trees mimics the behavior of pressure sensors attached to the heels and toes of a subject wearing instrumented shoes. However, an appropriate clinical assessment of gait requires the patients to walk barefoot, as the pronounced altering effects of shoes on gait parameters are well known, and have been clearly defined in a pediatric population [52]. Therefore, Gabel’s approach is unsuited for this specific clinical application in children, and this work proposes a framework based on a probabilistic matching of stride templates, with no shod feet requirements.

Other Kinect-based approaches include [53–58] but they are very limited. Sun et al. [53] uses an autoregressive moving average model with a Kalman filter for predicting the temporal series of the distances between Kinect and lower extremity markers. Gianaria et al. [55] and Staranowicz et al. [56] report simple methods for computing only the stride length and the walking speed. Pfister et al. [57] provides a way for estimating only the stride timing and two other body flexion parameters of a person on a treadmill. Auvinet et al. [58] focusses only on improving the accuracy of the heel strikes estimation of a person on a treadmill. Clark et al. [54] uses a very simple method for computing parameters, based on thresholding the local velocity of the foot and ankle joints. Those approaches have been tested with adults, and have never been subjected to the high degree of variability and noise typical of skeleton tracking sequences acquired from walking children. It is very difficult to cope with such severe conditions when relying on straight peak detection or thresholding. In contrast, the proposed approach performs a robust matching of probabilistic stride template models, allowing for accurate identification of heel strikes and toe-off instants. Also [59] uses templates for the step segmentation of signals collected from gyroscopes attached to instrumented shoes. However, their data is not vector valued, the templates are deterministic, and straight subsequence dynamic time warping [60] is used for template matching. Here, instead, the Kinect skeleton data is multidimensional, the templates are probabilistic, and the matching estimates jointly the global uniform temporal scaling [61], as well as the local non-uniform temporal scaling (under the form of dynamic time warping (DTW) [62]), of the templates, thus allowing for large adjustments in the length and shape of the detected strides. In particular, the approach brings together for the first time, probabilistic multidimensional uniform and non-uniform scaling with subsequence DTW techniques for computational efficiency.

Some previous Kinect methods have been compared against other systems. For instance, [41, 49, 54, 56–58] compare their approaches with the Vicon. However, only [54, 57] and [41] present a complete study of the concurrent validity of the methodology, while none of them are concerned with children’s gait analysis. Also in this work we validate the proposed approach by studying its concurrent validity against the GAITRite, which is a previously validated system even for children [15–17]. The GAITRite is very easy to setup and use with barefoot children, and has small space requirements.

The next section describes a computationally efficient algorithm we introduced for the temporal segmentation of data acquired by the Kinect, based on which a fully automated procedure for computing gait parameters is developed. This is described in the Methods section, along with a study conducted on healthy children for establishing the concurrent validity of the proposed approach.

## Temporal segmentation based on stride template models

In order to compute the gait parameters from a Microsoft Kinect observing a walking child, we analyze the raw skeleton tracking data it acquires. Specifically, as will become clearer in later sections, we need to automatically identify when each stride starts and ends. The estimation of such instants requires the design of a *temporal segmentation* algorithm that can cope with the high variability of the raw data, while being computationally efficient. This section introduces such algorithm, which will then be leveraged in the Methods section.

**Fig. 1:**
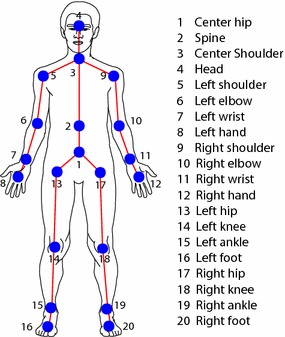
Skeleton. Graphical representation of the 20 joints composing the skeleton model used by the Kinect SDK for tracking the motion of a person

The raw tracking data acquired by the Kinect consists of a temporal sequence of length *n*, given by $\mathbf {x}_1, \ldots , \mathbf {x}_n$, or $\mathbf {x}_{1:n}$ for short, which is referred to as a *trial walk*. At time *t*, $\mathbf {x}_t \, =\, [ x_{1,t}; \ldots ; x_{20,t}] \in \mathbb {R}^{60}$ represents a *skeleton vector*, collecting the 3D positions of the 20 skeleton joints depicted in Fig. 1. The positions are assumed to be measured with respect to a *canonical reference frame*, which is attached to the walking child, and therefore is independent from the reference frame of the Kinect. The Methods section will explain how such reference frame can be computed automatically. In the sequel, the notations $\mathbf {x}_{\cdot : n}$, $\mathbf {x}_{1:\cdot }$, or $\mathbf {x}_{\cdot : \cdot }$, mean that the initial, final, or both time instants are not needed, or cannot be specified, depending on the context.

In order to automatically identify when a stride starts and ends, we take the approach of looking for the subsequence $\mathbf {x}_{t_s:t_e}$ (starting at $t_s$ and ending at $t_e$), of a trial walk $\mathbf {x}_{1:n}$, that best matches a *stride template model*

1 $$\begin{aligned} \mathcal {T}_m = (\varvec{\mu }_1, {\Lambda }_1), \cdots , (\varvec{\mu }_{m}, {\Lambda }_{m}), \end{aligned}$$

consisting of a sequence of $m\in \mathcal {M}$ pairs. Each pair $\varvec{\mu }_t$ and ${\Lambda }_t$ has the meaning of mean and covariance of a random vector that models the variability of the skeleton vector $\mathbf {x}_t$, at time *t* of a stride. The set $\mathcal {M}$ represents the possible temporal scales of the templates. Each scale *m* identifies a different template $\mathcal {T}_m$. The Methods section will explain how stride template models are learned from training data.

In the remaining part of this section we explain how we estimate $t_s$ and $t_e$ with different approaches. We begin with the simplest case where the template scale *m* is assumed to be known, then, we progressively improve the method by modeling uniform, and non-uniform temporal scaling, and finally we provide a computationally efficient approach that models both types of scaling variabilities.

### Constant stride time case

If the length of the strides was known to be *m*, the simplest way to find the subsequence $\mathbf {x}_{t_s:t_e}$ (where in this case $t_e = t_s+m-1$), that best matches a template $\mathcal {T}_m$, would be to look for the one (or equivalently, to look for $t_s$) that minimizes the distance

2 $$\begin{aligned} D_E(\mathbf {x}_{t_s: \cdot }, \mathcal {T}_m) = \sum _{i=1}^m \Vert \mathbf {x}_{t_s+i-1} - \varvec{\mu }_i \Vert ^2 \; , \end{aligned}$$

where $\Vert \cdot \Vert$ denotes the Euclidean norm. However, this approach would lead to a poor estimation due to the large amount of noise in the skeleton positions, and the large variability of joint trajectories across different subjects. Indeed, the Euclidean distance treats every joint position independently and in the same way, whereas the joints have different variances and are correlated. An approach that takes those issues into account entails modeling the likelihood probability distribution of a subsequence, given the stride template, $p(\mathbf {x}_{t_s:\cdot } | \mathcal {T}_m)$, and then estimating $t_s$ in the maximum likelihood (ML) sense. Given the statistical model for $\mathcal {T}_m$, this is equivalent to looking for $t_s$ that minimizes the distance

3 $$\begin{aligned} D_L(\mathbf {x}_{t_s:\cdot }, \mathcal {T}_m) = \sum _{i=1}^m (\mathbf {x}_{t_s+i-1} - \varvec{\mu }_i)^{\top } {\Lambda }_i^{-1}(\mathbf {x}_{t_s+i-1} - \varvec{\mu }_i) = \sum _{i=1}^m d_{M_i}(\mathbf {x}_{t_s+i-1}), \end{aligned}$$

where $d_{M_i}(\mathbf {x}_t)$ is the Mahalanobis distance of the skeleton vector $\mathbf {x}_t$, from the template component $\varvec{\mu }_i$, according to ${\Lambda }_i$.

### Uniform temporal scaling

Gait differences between different children correspond to skeleton trajectories exhibiting a variability in the uniform temporal scaling [61] (i.e., the global linear enlargement or shrinking of the time axis), such that relying on the assumption of a known equal length for $\mathcal {T}_m$, like in (3), will lead to inaccurate segmentations. This issue is addressed by augmenting (3) with the estimation of the amount of scaling to be applied. This is done by looking for the best matching subsequence $\mathbf {x}_{t_s:t_e}$ that minimizes the following ML *uniform scaling* distance

4 $$\begin{aligned} US_L(\mathbf {x}_{t_s:\cdot }, \mathcal {T}_{\cdot }) = \min _{m \in \mathcal {M}} \frac{1}{m} D_L(\mathbf {x}_{t_s:\cdot }, \mathcal {T}_m), \end{aligned}$$

where the factor 1/*m* has been introduced to make every scaling equally likely. This approach would provide the best templete size $\tilde{m}$, and time $t_s$.

### Non-uniform temporal scaling

Even after modeling uniform scaling, the residual temporal scaling variability, or so called non-uniform scaling, can still be significant to be modeled only by amplitude variation, like in (3). This is due to local variability of gait cycles in a person, to large amounts of noise in the joint trajectories, and to local variability of skeleton trajectories of children across different age groups. Non-uniform scaling can be handled by locally stretching the time axis, and dynamic time warping (DTW) [62] is known to be a good tool for doing so. DTW allows local flexibility in aligning time series, enabling the matching of sequences with tolerance of small local misalignments, thus achieving the goal of an accurate segmentation.

The ML estimation (3) can be augmented by modeling non-uniform scaling effects with DTW. To illustrate this, the *warping path*$p = (p_1, \cdots , p_w)$, where $p_l = (n_l, m_l)$, is introduced, which defines a mapping between the elements of two sequences. Assuming that *v* and *m* are the lengths of the sequences, then it must be that $p_1 = (1,1)$, $p_w = (v, m)$, $n_l \ge n_{l-1}$, $m_l \ge m_{l-1}$, and $\max (m,v) \le w \le m+v-1$. Therefore, the joint estimation of the non-uniform scaling and the ML subsequence $\mathbf {x}_{t_s : t_e}$ relies on minimizing the distance

5 $$\begin{aligned} DTW_L(\mathbf {x}_{t_s:t_e}, \mathcal {T}_m) = \min _{p} \sum _{l=1}^w d_{M_{m_l}}(\mathbf {x}_{t_s+n_l-1}), \end{aligned}$$

where, for each $t_s$ and $t_e$, *p* is optimized with dynamic programming, with complexity of *O*(*vm*) [62] with $v = t_e - t_s +1$, using this recursive definition of $DTW_L$

6 $$\begin{aligned} DTW_L( \varnothing , \varnothing )&= 0 \nonumber \\ DTW_L(\mathbf {x}_{t_s:t_e}, \varnothing )&= DTW_L( \varnothing , \mathcal {T}_m) = \infty \nonumber \\ DTW_L(\mathbf {x}_{t_s:t_e}, \mathcal {T}_m)&= d_{M_{m}}(\mathbf {x}_{t_e}) + \min \left\{ \begin{array}{l} DTW_L(\mathbf {x}_{t_s:t_{e-1}}, \mathcal {T}_m) \nonumber\\ DTW_L(\mathbf {x}_{t_s:t_e}, \mathcal {T}_{m,m-1} ) \\ DTW_L(\mathbf {x}_{t_s:t_{e-1}}, \mathcal {T}_{m,m-1} ) \\ \end{array} \right. \end{aligned}$$

In (6) the notation $\mathcal {T}_{m,i}$ indicates the subsequence of $\mathcal {T}_m$ up to the *i*-th pair.

### Joint uniform and non-uniform scaling

The framework expected to provide the best segmentation accuracy combines the ML estimation of a subsequence with the uniform and non-uniform scaling. This is done by replacing $D_L$ with $DTW_L$ in (4), which gives an extension of the criterion used in [63], here referred to as ML *scaling and time warping matching (SWM)*, which estimates the matching subsequence $\mathbf {x}_{t_s:t_e}$ that minimizes the following distance

7 $$\begin{aligned} SWM_L(\mathbf {x}_{t_s:t_e}, \mathcal {T}_{\cdot }) = \min _{m \in \mathcal {M}} \frac{1}{m} DTW_L(\mathbf {x}_{t_s:t_e}, \mathcal {T}_m). \end{aligned}$$

Besides $t_s$ and $t_e$, this approach provides also the optimal template size $m^*$.

The computational complexity analysis with respect to *m* and *n* provides insights on the criterions described so far. In particular, finding the matching subsequence with (2) or (3) implies testing for every $t_s$, which requires *O*(*n*) operations. $US_L$ (4), requires testing for every $t_s$ and for all the $| \mathcal {M} |$ templates, leading to $O(n|\mathcal {M}|)$ operations. $DTW_L$ (5), requires *O*(*vm*) operations, but a subsequence is found by testing every combination of $t_s$ and $t_e$, requiring a total of $O(n^3m)$ operations. Finally, for every pair of $t_s$ and $t_e$, $SWM_L$ tests $| \mathcal {M} |$ different templates, leading to a complexity of $O(n^3 m |\mathcal {M}|)$. Therefore, (7) leads to the highest computational complexity, which can quickly become impractical as soon as the length of the trial walk increases or the dependency from *m* and $|\mathcal {M}|$ is not kept under control.

### Efficient joint uniform and non-uniform scaling

Here the computational efficiency of (7) is improved by exploiting subsequence matching techniques, which do not require testing for every pair $t_s$ and $t_e$. Those include a subsequence DTW (SDTW) approach [60, 64], which computes the warping path *p*, the starting and ending times $t_s$ and $t_e$, and the DTW distance of the best matching subsequence. The ML extension of SDTW, indicated with $SDTW_L$, is computed by solving the following recursion

8 $$\begin{aligned} D_S( \varnothing , \varnothing )&= D_S(\mathbf {x}_{\cdot :t}, \varnothing ) = 0 \\ D_S( \varnothing , \mathcal {T}_{m,i})&= \infty \nonumber \\ D_S(\mathbf {x}_{\cdot :t}, \mathcal {T}_{m,i})&= d_{M_{i}}(\mathbf {x}_{t}) + \min \left\{ \begin{array}{l} D_S(\mathbf {x}_{\cdot :t-1}, \mathcal {T}_{m,i} )\nonumber \\ D_S(\mathbf {x}_{\cdot :t}, \mathcal {T}_{m,i-1}) \\ D_S(\mathbf {x}_{\cdot :t-1}, \mathcal {T}_{m,i-1} ) \\ \end{array} \right. \nonumber \\SDTW_L(\mathbf {x}_{\cdot : \cdot }, \mathcal {T}_m )& = \min _t D_S ( \mathbf {x}_{\cdot : t }, \mathcal {T}_m ), \nonumber \end{aligned}$$

where $D_S(\mathbf {x}_{\cdot :t}, \mathcal {T}_{m,i})$ is a matrix, storing the cost accumulated so far, by the best warping path that includes the mapping element (*t*, *i*). Equation (8) is solved with dynamic programming, with a complexity of *O*(*nm*) [64]. Compared with minimizing $DTW_L$ and checking for every pair of $t_s$ and $t_e$, the complexity has improved by a factor of $n^2$, which is remarkable.

The efficiency of computing the best matching subsequence $\mathbf {x}_{t_s:t_e}$ through (7) improves greatly by replacing $DTW_L$ with $SDTW_L$, leading to the new ML *subsequence scaling and time warping matching (SSWM)* criterion, given by

9 $$\begin{aligned} SSWM_L(\mathbf {x}_{\cdot :\cdot }, \mathcal {T}_{\cdot }) = \min _{m \in \mathcal {M}} \frac{1}{m} SDTW_L(\mathbf {x}_{\cdot :\cdot }, \mathcal {T}_m). \end{aligned}$$

If $m^*$ is the optimal stride template size provided by (9) (which is supposed to be equal to the one provided by (7)), then, according to SDTW [60, 64], $t_e$ is given by

10 $$\begin{aligned} t_e = \arg \min _t D_S ( \mathbf {x}_{\cdot : t }, \mathcal {T}_{m^*} ). \end{aligned}$$

While computing the recursion (8), a warping matrix is populated, which allows tracing the path *p* from the end $p_w = (t_e,m^*)$, back to the beginning $p_1 = (t_s,1)$, from which $t_s$ is readily available. The fundamental advantage of using (9) versus (7) is that the computational complexity of $SSWM_L$ is $O(nm|\mathcal {M}|)$, which improves by a factor of $n^2$ against $SWM_L$, enabling the implementation of the approach on a low-cost platform with limited computing power.

## Methods

This section leverages the technique we developed previously, and introduces a fully automatic system for gait analysis based on the Kinect. The system is also validated against the GAITRite with a study conducted on healthy children. This is the first time the Kinect is validated for children’s gait analysis in healthy subjects. The validation process requires simultaneous measurements of gait parameters to be acquired by a previously validated tool that acts as the criterion (the GAITRite), and by the new system to be validated (based on the Kinect). The chosen criterion is particularly well suited to work with children, and does not interfere with the Kinect acquisitions. The remaining of the section describes the details of the study and of the new gait analysis system.

### Materials: GAITRite

A GAITRite system (v3.9 [19]) was used. It consists of an electronic roll-up walkway connected to a laptop computer with a USB interface cable. The walkway is approximately 520 cm long, with an active sensor area that is 427 cm long and 61 cm wide, containing 16,128 pressure sensors arranged in a grid pattern with a spatial resolution of 1.27 cm. Data from the activated sensors is collected and transferred to the personal computer through a serial port connection. The sampling frequency of the system is 80 Hz.

### Materials: Kinect

The Microsoft Kinect is a sensing device designed to allow controller-free game play on the Microsoft Xbox. Here the first generation of Kinect was used [19] , also known as Kinect for Xbox 360, or sometimes Kinect v1. The sensor contains an RGB as well as an infrared (IR) camera and an IR light emitter. The emitter projects a known pattern onto the scene, based on which the pixel intensities of the images captured by the IR camera are decoded into depth distances. Therefore, the Kinect captures standard video data, as well as depth data at 30 frames per second, encoded in an 11-bit image with resolution of $640\times 480$ pixels. The Kinect SDK, of which the version 1.5 was used, gives access to the raw RGB and depth data, and also to a 3D virtual skeleton of the body of the people appearing in the scene [20]. See Fig.  1. The SDK maintains skeleton tracking at video rate, within a depth range that can stretch over a range of approximately 0.7–6 m.

### Experimental setup

**Fig. 2:**
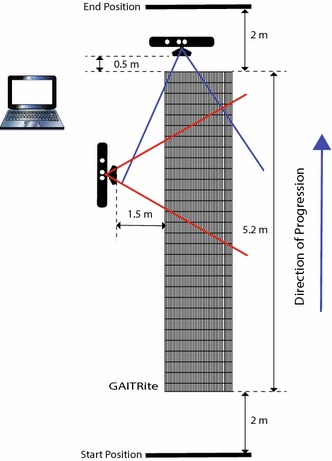
Experimental setup. Layout of the GAITRite walkway with the position and field of views of the front-view and side-view Kinects. The front-view Kinect performs the fitting and tracking of a skeleton model composed of 20 joints, depicted in Fig. 1

The setup of the GAITRite and two Kinect sensors is depicted in Fig. 2. In order to allow the subjects to perform a full walkthrough of the walkway with a free exit, the front-view Kinect was placed at the end of the GAITRite and closer to one of the corners. Moreover, it was positioned 0.5 m from the walkway edge to allow for a high overlap of its tracking range with the walkway extension. The second Kinect was looking at the walkway from the side. It was positioned approximately 1.5 m from the side walkway edge. Its purpose was to provide data for future use, and for supporting the manual annotation of the heel strikes and toe-off instants, as will be explained later. However, we stress the fact that the side-view Kinect was not used for 3D skeleton tracking. Only the front-view Kinect was devoted to that purpose. So, the side-view Kinect is used only for providing a better data visualization during the annotation phase, and the gait analysis is performed solely with data collected by the front-view Kinect. Both Kinects were mounted on tripods at a height of 1.3 m.

### Subjects

Following the West Virginia University Institutional Review Board approval, 25 child subjects (15 females and 10 males) were recruited to participe in a data collection study. Those were healthy children with no known gait abnormalities. Their average age ($\pm$ standard deviation) was $3.26 \pm 0.96$ years, with a range from 2 to 4 years. Their average leg length was $43.15\pm 5.64$ cm. They appeared for the collection at the Pediatric and Adolescent Group Practice of the Physician Office Center of the West Virginia University Hospitals. Written informed consent was obtained from the parents of each subject prior to data collection.

### Experimental protocol

For every subject the data collection began with the acquisition of anthropometric measurements such as leg length, which is required by the GAITRite software. Subjects were instructed to walk barefoot over the GAITRite mat, at his or her usual comfortable walking speed, and they were given the opportunity to perform practice walks to familiarize with the procedure. In order to minimize the acceleration and deceleration effects, the subjects started the waking trials 2 m before and finished 2 m after the mat. At least three trials were recorded for each subject, in order to aggregate enough step cycles captured by the front-view Kinect for the computation of the gait parameters. The data recording from the GAITRite and the two Kinects was performed simultaneously by a single laptop workstation. In particular, we developed an application capable of recording temporally synchronized data streams coming from the front-view and side-view Kinects. However, skeleton tracking was performed by, and recorded from, only the front-view Kinect.

### Gait parameters

**Fig. 3:**
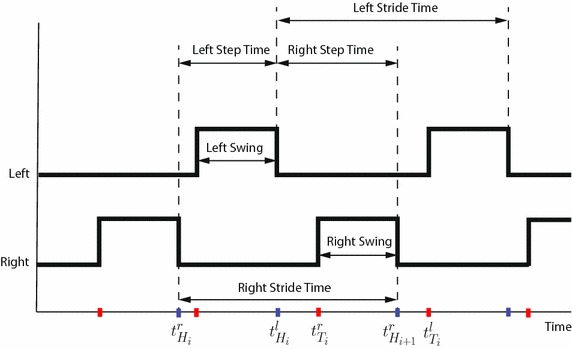
Temporal parameters. Summary of the definitions of the temporal gait parameters. A low signal represents a foot touching the ground, and a high signal means it is not touching. Ascending (*red*) and descending (*blue*) fronts identify toe-off and heel strike instants, respectively

The GAITRite computes a number of temporal and spatial gait parameters. Figure  3 summarizes the definitions of the *temporal parameters*. In particular, with respect to the *i*-th stride cycle of the right foot, for a subject with a gait with no abnormalities, $t_{H_i}^r$ represents the time that the mat first senses the right heel, so it is the right *heel strike* first contact. Similarly, $t_{H_i}^l$ is the left heel strike first contact. Moreover, $t_{T_i}^r$ represents the time that the mat stops sensing the right forefoot, so it is the right *toe-off* last contact. Similarly, $t_{T_i}^l$ is the left toe-off last contact. Unless otherwise specified, those quantities are always measured in seconds, and from them it is possible to compute several temporal parameters. This work has considered the ones defined below.

The *step time*, *S*, is the time elapsed from the heel strike of one foot to the heel strike of the opposite foot. If *k* stride cycles are available, for the right foot, $S^r$ is computed as

11 $$\begin{aligned} S^r = \frac{1}{k} \sum _{i=1}^k (t_{H_{i+1}}^r - t_{H_i}^l). \end{aligned}$$

The *stride time*, *R*, is the time elapsed from the heel strikes of two consecutive footfalls of the same foot. If *k* right stride cycles are available, $R^r$ is computed as

12 $$\begin{aligned} R^r = \frac{1}{k} \sum _{i=1}^k (t_{H_{i+1}}^r - t_{H_i}^r). \end{aligned}$$

The number of strides taken in one minute is referred to as *cadence*, which is given by $C \, = \, 60/R^r+60/R^l$.

The *swing time*, *W*, is the time elapsed between the toe-off of the current footfall to the heel strike of the next footfall of the same foot. If *k* right stride cycles are available, $W^r$ is given by

13 $$\begin{aligned} W^r = \frac{1}{k} \sum _{i=1}^k (t_{H_{i+1}}^r - t_{T_{i}}^r). \end{aligned}$$

**Fig. 4:**
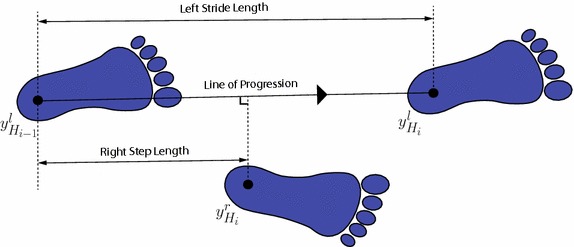
Spatial parameters. Summary of the definitions of the spatial gait parameters, based on the geometric position of the heel centers

The GAITRite computes also a number of *spatial parameters*. Many of them rely on the position of the *heel centers*$y_{H_i}$, estimated from the footprint revealed by the pressure sensors when the foot is flat and touching the mat (see Fig. 4). This work has considered the spatial gait parameters defined below, which are based on the heel center positions, where unless otherwise specified, every length is measured in centimeters.

The *stride length*, *L*, is the distance between the heel centers of two consecutive footprints of the same foot. For instance, if *k* right stride cycles are available, $L^r$ is computed as

14 $$\begin{aligned} L^r = \frac{1}{k} \sum _{i=1}^k \Vert y_{H_{i+1}}^r - y_{H_{i}}^r \Vert . \end{aligned}$$

Given the stride length and the stride time, the average *velocity*, *V*, is computed as the average stride length divided by the average stride time, i.e., $V=(L^r+L^l)/(R^r+R^l)$.

The *step length*, *D*, requires the *line of progression*, which is defined by the segment obtained by connecting the heel centers of two consecutive footprints of the same foot, e.g., $y_{H_{i-1}}^l$ and $y_{H_{i}}^l$ (see Fig. 4). Then, the step length of the right foot is the distance between $y_{H_{i-1}}^l$ and the projection of $y_{H_i}^r$ on the line of progression. Analytically, when *k* right stride cycles are available, $D^r$ is given by

15 $$\begin{aligned} D^r = \frac{1}{k} \sum _{i=1}^k \frac{ ( y_{H_{i}}^r - y_{H_{i-1}}^l )^{\top } ( y_{H_{i}}^l - y_{H_{i-1}}^l )}{\Vert y_{H_{i}}^l - y_{H_{i-1}}^l \Vert } . \end{aligned}$$

Finally, although the parameters have been introduced for the right foot (superscript *r*), they are also valid for the left foot with a careful substitution of the superscripts (from *r* to *l*) and adjustment of the indices. Moreover, all the parameters could be averaged among right and left foot, besides being computed for each of them separately.

### Extraction of gait parameters with GAITRite

From the recorded spatio-temporal occurrence of footprints, the proprietary GAITRite software automatically computes the heel strikes, the toe-offs, and other temporal instants, as well as the heel centers and other geometric properties of the footprints. Those are then used for computing several gait parameters, including those defined in the previous section.

### Manual extraction of gait parameters from Kinect data

**Fig. 5:**
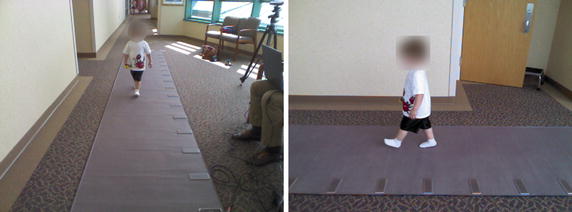
Kinect views. Two frames captured by the RGB cameras of the front-view Kinect (*left*) and the side-view Kinect (*right*) during a trial walk

**Fig. 6:**
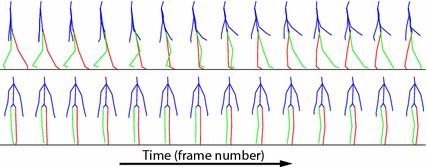
Skeleton data. Fraction of a skeleton time series $\mathbf {y}_{1:n}$, including a right swing cycle acquired with the Microsoft Kinect. The body parts are shown in *blue*, the left leg in *red*, and the right leg in *green*. The data was acquired with the front-view Kinect

An annotation tool was developed to visualize the data acquired during trial walks, and to allow a human annotator to conveniently record the video frame numbers corresponding to the time instants of the heel strikes $\tilde{t}_{H_i}$, and the toe-offs $\tilde{t}_{T_i}$. The tool was developed using Matlab, and allows opening, visualizing and scrolling through three streams of data at the same time. Those streams correspond to (a) the RGB data coming from the front-view Kinect (see left of Fig. 5), (b) the RGB data coming from the side-view Kinect (see right of Fig. 5), and (c) the skeleton data coming from the front-view Kinect (see Fig. 6). Therefore, for a given frame number *t*, the annotation tool shows three views, corresponding to (a), (b), and (c). The user can scroll through the time axis back and forth using the arrow keys. Doing so increases and decreases the frame number *t*, and the three data views change accordingly. The tool allows the user to quickly label specific frame numbers as *right/left toe-off*, or as *right/left heel strike*. This functionality is used by a human annotator that carefully observes the three views (a), (b), and (c), and visually identifies and labels the frame numbers corresponding to heel strikes and toe-off instants. After annotating the entire dataset, we realized that having the side-view was very helpful. On the other hand, we found the skeleton view less useful, since the data appeared to be too noisy to accurately assess visually the occurrence of heel strikes and toe-offs.

The annotation process produces a set of pairs $\{(\tilde{t}_{H_i}, \tilde{t}_{T_i})\}$ that can be used for computing the temporal parameters defined previously. The spatial parameters, instead, require the heel center positions, which are estimated as follows. Let *y*(*t*) indicate the 3D coordinates at time *t*, of a point attached to a foot such that at foot flat $y(t) = y_{H_i}$, i.e. *y*(*t*) is the heel center position when the foot is flat. Notice that the position of *y* at heel strike, $y( t_{H_i} )$, and at foot flat, $y_{H_i}$, are almost the same. In addition, $y( t_{H_i} )$ can be approximated by the coordinates of the closest skeleton joint, which is the ankle, given by $y_{a,t_{H_i}}$. Therefore, spatial parameters are estimated with the heel centers $\{\tilde{y}_{{H_i}} \}$, computed by approximating $\tilde{y}_{{H_i}}$ with $y_{a,t_{H_i}}$. This has limited impact on the parameters, because they entail computing distances between heel centers at foot flat, which are almost identical to distances between the same foot points at heel strike. Finally, we will show later that the set $\{(\tilde{t}_{H_i}, \tilde{t}_{T_i})\}$ is used also as training labels for learning the stride template models.

### Automatic extraction of gait parameters from Kinect data

Given Kinect skeleton tracking data, this section introduces a fully automated approach for estimating the heel strike and toe-off instants, as well as the heel centers, from which temporal and spatial gait parameters can be computed. For a trial walk of length *n*, such tracking data is given by $\mathbf {y}_1, \cdots , \mathbf {y}_n$, or $\mathbf {y}_{1:n}$ for short. At time *t*, $\mathbf {y}_t \, = \, [ y_{1,t}; \cdots ; y_{20,t}] \in \mathbb {R}^{60}$ represents a *skeleton vector*, collecting the 3D positions of the 20 skeleton joints, with respect to the Kinect reference frame.

Estimating the heel strike and toe-off instants entails the temporal segmentation of the trial walk $\mathbf {y}_{1:n}$, which could be attained with the automatic procedure described in the previous section, by finding the subsequences of $\mathbf {y}_{1:n}$ that match the template models. However, this idea cannot be directly applied, unless we first design the following: (a) a procedure for mapping trial walk data, expressed with respect to the Kinect reference frame, onto data expressed with respect to the *canonical reference frame*, where the stride templates are defined; (b) a procedure for learning the stride templates; (c) a robust temporal segmentation that identified all the heel strike and toe-off instants. The following sections will address those steps, and also the final step of estimating the heel centers.

#### Canonical reference frame

**Fig. 7:**
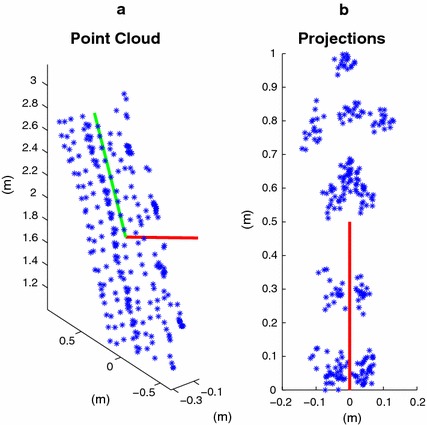
Skeleton point cloud. **a** point could of the 3D joint positions, downsampled for visualization purposes. Each *blue asterisk* is a point. The *green line* is the first principal component (p.c.) of the cloud. The *red line* is the second p.c. **b** Point cloud projected onto the plane perpendicular to the first p.c. The second p.c. (*red line*) indicates the direction normal to the ground plane

From $\mathbf {y}_{1:n}$ a *canonical reference frame*, independent from the Kinect reference frame and robust to noise, is estimated as follows. All the joint positions $\{ y_{i,t} \}$ are collected into a matrix $Y = [y_{1,1}, y_{2,1}, \cdots ]$, and treated as a point cloud. After removing the mean from *Y*, the principal components are computed via singular value decomposition (SVD) [65]. The first principal component (p.c.) is parallel to the ground plane, and identifies the average direction of progression (green line in Fig. 7a). This is because the cloud is elongated in the walking direction of the subject and is typically extending for more than 3 m along a roughly straight line. The second p.c., instead, is perpendicular to the ground plane (red lines in Fig. 7). This is because the projection of the cloud onto the plane perpendicular to the first p.c. appears elongated towards the vertical extension of the body of a subject, which is always greater than the horizontal, and enjoys the right-left symmetry. See Fig. 7b. The second p.c., oriented towards the outside of the ground floor is the first axis $u_1$, of the canonical reference frame. This method is quite robust to large amounts of noise and tracking errors. In addition, the joints corresponding to hands, wrists, and elbows are removed from *Y* to make the estimation of $u_1$ robust to unusual and asymmetric arm movements during a trial walk.

At time *t*, the second axis $u_{2,t}$ of the canonical reference frame, points along the current direction of progression of the subject, and is computed as follows. From $\mathbf {y}_{t}$, a skeleton center point $y_{c,t}$ is computed by averaging the joints given by the right hip, the left hip, and the center hip. Thus, the point cloud $[y_{c,t-\tau }$, $\cdots$, $y_{c,t+\tau }]$ is elongated in the current direction of progression, which can be computed via SVD after removing the mean of the cloud. In particular, $u_{2,t}$ is computed from the first singular vector, after orienting it in the direction of progression of the subject, projecting it onto the ground plane defined by $u_1$, and setting its norm to 1. The third axis is simply computed by the cross product $u_{3,t} \, = \, u_1 \times u_{2,t}$.

Finally, the origin of the canonical reference frame must be independent from the origin of the Kinect reference frame, and it is defined as the projection of the skeleton center point $y_{c,t}$ onto the ground plane. Therefore, to map $\mathbf {y}_t$ onto $\mathbf {x}_t { = } [ x_{1,t}; \cdots ; x_{20,t}] \in \mathbb {R}^{60}$, where every joint position $x_{i,t}$ is expressed in the canonical reference frame, let us define $U_t = [ u_1, u_{2,t}, u_{3,t} ] \in \mathbb {R}^{3 \times 3}$, and let $y_{0,t}$ be the lowest joint of $\mathbf {y}_t$ along $u_1$, which is touching the ground plane. Then, $x_{i,t}$ is related to $y_{i,t}$ as follows

16 $$\begin{aligned} x_{i,t} = U_t^{\top } y_{i,t} - \left[ \begin{array}{c} u_1^{\top } y_{0,t} \\ u_{2,t}^{\top } y_{c,t} \\ u_{3,t}^{\top } y_{c,t} \end{array} \right]. \end{aligned}$$

We stress the fact that mapping the trial walk onto the canonical reference frame is a fully automatic process, and that the entire gait analysis framework never requires any form of (intrinsic or extrinsic) calibration of the Kinect. Also, the mapping assumes that a trial walk occurs roughly on a straight line, regardless of whether the Kinect is strictly in frontal position, as long as the skeleton tracking can be performed with sufficient accuracy. Finally, large deviations from a straight trial walk trajectory could be handled with a more complex mapping procedure, which is beyond the scope of this work.

#### Learning the stride template models

From each training trial walk $\mathbf {x}_{1:n}$, using the heel strike annotations obtained manually, the subsequences representing single stride cycles are extracted. If $\overline{m}$ and $\sigma$ are the rounded mean and standard deviation of the lengths of the subsequences, template models are learned for each integer dimension $m\in \mathcal {M}$, where $\mathcal {M} = \{ \overline{m} -2 \sigma , \overline{m} - 2 \sigma +1, \cdots , \overline{m} + 2 \sigma \}$. This guarantees that about 95 % of strides will have a length in the range covered by $\mathcal {M}$. For a dimension *m*, the subsequences are resampled to a length *m* with spline interpolation, and divided into the sets of right and left strides. For each set and time instant the mean and covariance are computed, generating the *right* and *left stride template models*

17 $$\begin{aligned} \mathcal {T}_m^r =&\, (\varvec{\mu }_1^r, {\Lambda }_1^r), \cdots , (\varvec{\mu }_{m}^r, {\Lambda }_{m}^r) \nonumber \\ \mathcal {T}_m^l =&\, (\varvec{\mu }_1^l, {\Lambda }_1^l), \cdots , (\varvec{\mu }_{m}^l, {\Lambda }_{m}^l). \end{aligned}$$

**Fig. 8:**
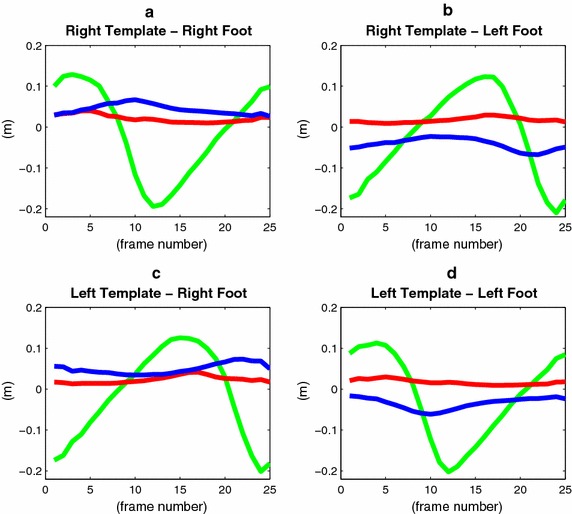
Stride templates. Plots of the means of the ankle joint positions of the *right* (**a**, **b**), and *left* (**c**, **d**) templates. The coordinates along the $u_1$, $u_2$, and $u_3$ axes are shown in *red*, *green*, and *blue*, respectively. The left template is essentially the right template circular-shifted by the left step time. They have been learned from different data sets, and show minor differences

Throughout the paper, the superscripts *r* and *l* are used only when indicating right or left is strictly needed. Figure  8 shows the plots of the means of the stride templates for the ankle joints. Within a stride template, there is a time index corresponding to the toe-off $t_T$. This is computed by averaging the toe-off annotations obtained after having resampled the stride cycle subsequences to a length *m*.

Finally, we note that learning the stride template models is a data-driven process that needs to be performed only once. This means that a user of the proposed gait analysis approach would not need to collect data, perform annotations, and learn the stride models, because they would be given to him already as part of the system.

#### Temporal segmentation

Given a test trial walk $\mathbf {x}_{1:n}$ and the stride templates (17), *computing the temporal segmentation entails estimating how many right and left stride cycles are present, and when each of them starts and ends*. This will tell where the heel strike and toe-off instants are located.

**Fig. 9:**
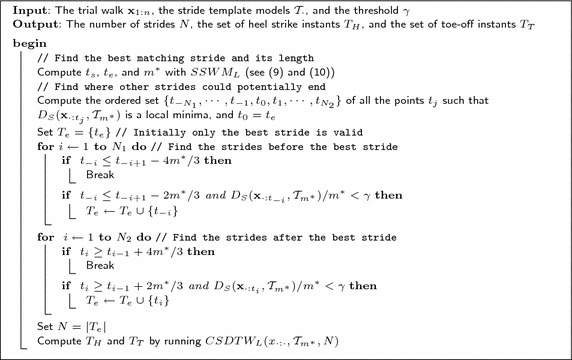
TrialWalkSegmentation Algorithm. Algoritm that summarizes the steps necessary for segmenting a trial walk $\mathbf {x}_{1:n}$, into strides delimited by heel strike instants $T_H$, and toe-off instants $T_T$. The TrialWalkSegmentation algorithm has to be executed with the right (*left*) stride template models to estimate the right (*left*) stride segmentation instants

After estimating the subsequence $\mathbf {x}_{t_s:t_e}$ that best matches a stride template according to (9) and (10), other subsequences, supposedly corresponding to additional stride cycles, are estimated by examining the other local minima of $D_S ( \mathbf {x}_{\cdot : t }, \mathcal {T}_{m^*} )$. In particular, a time $t_{e_i}$ of a local minima $D_S ( \mathbf {x}_{\cdot : t_{e_i} }, \mathcal {T}_{m^*} )$ is accepted as the ending time of the *i*-th stride if $t_{e_i} \le t_{e_{j}} -2m^*/3$, and $t_{e_i} \ge t_{e_{j}} +2m^*/3$, and if $D_S ( \mathbf {x}_{\cdot : t_{e_i} }, \mathcal {T}_{m^*} )/m^* < \gamma$. This ensures that $t_{e_i}$ is sufficiently far away from the ending times observed so far, $\{ t_{e_j} \}$, and that the normalized DTW distance of the subsequence from the template $\mathcal {T}_{m^*}$ is below a given threshold $\gamma$. In addition, ending times are sequentially accepted by searching for minima in directions expanding from the initial ending time. This makes the subsequences correspond to contiguous strides. Ending times are no longer accepted if $t_{e_i} \le t_{e_j} -4 m^*/3$, or $t_{e_i} \ge t_{e_j} +4 m^*/3$, assuming that the search was expanding in the decreasing or increasing time direction, respectively, and $t_{e_j}$ is the ending time at the boundary of the expansion. The number *N* of accepted ending times $T_e = \{ t_{e_j} \}$ is the number of stride cycles found in the trial walk. Figure 9 summarizes the temporal segmentation procedure, named TrialWalkSegmentation, which includes the estimation of contiguous strides as explained next. The algorithm has to be repeated twice: once for the right and once for the left foot.

**Fig. 10:**
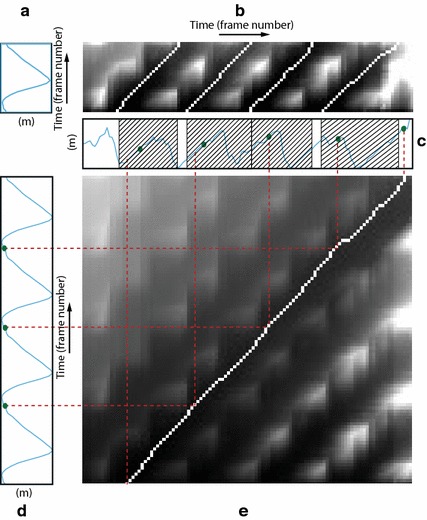
Contiguous estimation of time instants. **a** Second coordinate of the right ankle, extracted from $\mathcal {T}_{m^*}^r$. **b** Accumulated cost matrix $D_S ( \mathbf {x}_{\cdot :t}, \mathcal {T}_{m^*}^r)$. Four local minima along the top edge identify the ending times of four matching subsequences. Four traced-back paths identify the starting times. **c** Second coordinate of the right ankle extracted from $\mathbf {x}_{1:n}$. $N=4$ right strides with length $m^*$ are identified, and two gaps between matching subsequences are formed. The green dots represent the ground-truth segmentation. **d** Second coordinate of the right ankle, extracted from the concatenation of four templates $\bigoplus _{i=1}^4 \mathcal {T}_{m^*}^r$. **e** Accumulated cost matrix $D_S ( \mathbf {x}_{\cdot :t}, \bigoplus _{i=1}^4 \mathcal {T}_{m^*}^r)$. The minimum along the top edge identifies the ending time of four right strides. The traced-back path identifies the starting time. Heel strike and toe-off instants are identified by mapping them from the time domain of the concatenated templates (**d**), to the time domain of the trial walk (**c**), according to the warping path (*red lines*)

*Heel strike and toe-off instants* The *N* identified subsequences are not guaranteed to be “perfectly” contiguous, whereas for consecutive strides of the same foot it should be that $t_{s_{i+1}} = t_{e_{i}}+1$. This can be ensured by composing a new template model by concatenating *N* templates $\mathcal {T}_{m^*} \oplus \cdots \oplus \mathcal {T}_{m^*}$ and matching it against the trial walk by computing $SDTW_L(\mathbf {x}_{\cdot :\cdot }, \mathcal {T}_{m^*} \oplus \cdots \oplus \mathcal {T}_{m^*} )$. The set of heel strikes $T_H = \{ t_{H_i} \}$ is obtained by mapping, through the estimated warping path, the beginning of each template onto the trial walk. Similarly, the set of toe-off instants $T_T =\{ t_{T_i} \}$ is estimated by mapping the toe-off instants of each template. This procedure, indicated as *contiguous*$SDTW_L$, or $CSDTW_L$, is depicted in Fig. 10 and allows a very precise contiguous estimation of the heel-strikes and toe-offs for each foot.

*Heel centers* The heel centers are estimated by projecting the ankle joint positions onto the ground plane at the heel strike instants $\{ t_{H_i} \}$. Therefore, if $y_{0,t_{H_i}}$ are the coordinates of a skeleton point touching the ground plane at time $t_{H_i}$, and $y_{a,t_{H_i}}$ are the coordinates of an ankle joint at the same time, then the corresponding heel center coordinates, expressed in the Kinect reference frame, are given by

18 $$\begin{aligned} y_{H_i} = U_{t_{H_i}} \left[ \begin{array}{c} u_1^{\top } y_{0,t_{H_i}} \\ u_{2,t_{H_i}}^{\top } y_{a,t_{H_i}} \\ u_{3,t_{H_i}}^{\top } y_{a,t_{H_i}} \end{array} \right]. \end{aligned}$$

### Statistical analysis

For any given subject, step-by-step gait parameters computed from all the trial walks were averaged. Means and standard deviations (SD) for the system to be validated and the criterion were calculated. Bland and Altman plots were generated to provide a visual representation of the heteroscedasticity of the data [66]. The normal distribution of the data was tested with a Kolmogorov-Smirnov test. Agreement between the average parameters from the Kinect and GAITRite devices were assessed using Bland-Altman bias and limits of agreement (LoA), computed according to [67], Pearson’s correlation ($\rho$) [68], the concordance correlation coefficient (CCC) [69, 70], and intra-class correlation (ICC) [71]. Pearson’s correlation and CCC assess the relative and overall agreement, respectively, between the two methods. In particular, while the Pearson’s correlation focusses on precision, CCC assesses both precision and deviation from the line of identity (accuracy). A visual representation of this assessment is provided also by the associated scatter plots. ICC coefficients of the type (2, *k*) with absolute agreement (as previously reported in [12, 72]), were used to further evaluate the level of agreement between methods. A repeatability analysis for the Kinect is performed by computing gait parameters as averages out of single trial walks. Repeatability coefficients are computed by considering pairs of trial walks from the same subject, and are expressed in absolute value (as 2 times the SD [66]), as well as in a percentage of the mean.

## Results

### Automatic estimation

The approach is evaluated with a leave-one-subject-out cross-validation approach. This means that the trial walks of each subject are processed with the template models learned from the trial walks of all the remaining subjects. The manual estimates of the heel strike and toe-off instants are used as labels for learning the templates, and for performance evaluation of the automatic segmentation. The average length of a stride is $\overline{m} = 25$ frames, the template models are learned for each dimension *m* in the range [15, 35], and $\tau$ is set to 3.

The automatic trial walk segmentation is evaluated by computing the Rand index [73] and the accuracy on detection (AoD) [74], which here is defined as follows. Let $\mathbf {t} = [t_s,t_e]$ indicate the support of a subsequence $\mathbf {x}_{t_s:t_e}$, and let $\mathbf {g} = [g_s, g_e]$ indicate the corresponding ground-truth support. The percentage of overlap between the supports is defined as

19 $$\begin{aligned} P_{\mathbf {t},\mathbf {g}} = \frac{\min \{t_e,g_e\} - \max \{t_s,g_s\} + 1}{\max \{t_e,g_e\} - \min \{t_s,g_s\} + 1} \; , \end{aligned}$$

when $\min \{t_e,g_e\} \ge \max \{t_s,g_s\}$, otherwise $P_{\mathbf {t},\mathbf {g}} = 0$. AoD is the average overlapping percentage. If $\mathcal {P} = \{ P_{\mathbf {t},\mathbf {g}} \}$ is the set of all the overlapping percentages, then $\text {AoD} = 1/|\mathcal {P}| \sum _{P \in \mathcal {P}} P$. While the Rand index and the AoD measure the accuracy of the temporal segmentation, the standard deviation of the estimation error $t_{\cdot }-g_{\cdot }$, where $t_{\cdot }$ and $g_{\cdot }$ are corresponding starting or ending times, is indicative of the precision of the instant estimates and is also computed.

Table 1 reports the AoD, the Rand index, and the SD of the instant estimation error for several approaches. For a trial walk, $US_L$ provides a template length $\tilde{m}$, which is used to estimate the following non-overlapping subsequences in a greedy fashion by minimizing $D_M(\mathbf {x}_{t_s:\cdot }, \mathcal {T}_{\tilde{m}})$. $SDTW_L$ segments the same trial walk with templates of length $\tilde{m}$. The fourth row of Table 1 corresponds to using $CSDTW_L$ with the template length set to $\tilde{m}$. $SSWM_L$, instead, provides the optimal template size $m^*$, for any given trial walk, which is also used by $CSDTW_L$ in the last row of Table 1. By all metrics, $SSWM_L$ is the best approach for proposing the optimal template size, $m^*$, and number of strides, *N*, to be used in the contiguous refinement $CSDTW_L$. Thus, the combination of $SSWM_L$ and $CSDTW_L$ represents the automatic segmentation method of choice, and is referred to as Kinect-A. Finally, in all experiments, $\gamma$ was set to 1.

**Table 1:** Temporal segmentation

Method	AoD	Rand index	Error SD (s)
$US_L$	0.722	0.745	0.083
$SDTW_L$ with $\tilde{m}$	0.825	0.838	0.068
$SSWM_L$	0.893	0.901	0.059
$CSDTW_L$ with $\tilde{m}$	0.882	0.892	0.060
$CSDTW_L$ with $m^*$	0.913	0.928	0.055

Comparison between the accuracy and precision of several temporal segmentation methods

Figure  10 shows how Kinect-A computes the heel strike and toe-off instants in two steps. The first one is summarized by Fig. 10a–c, where $SSWM_L$ computes the optimal length $m^*$, and *N* subsequences potentially separated by gaps. The second step is summarized by Fig.  10c–e, where $CSDTW_L$ with parameters $m^*$ and *N*, computes *N* contiguous stride subsequences. The green dots represent the ground-truth segmentation. The final segmentation, defined by the red lines, shows qualitatively a clear improvement with respect to the initial segmentation with gaps. Note that Fig. 10a, c, d only show the plots of one coordinate component of the ankle joint. However, the algorithms use the coordinates of all the leg joints and the center hip joint. For a typical trial walk, the number of contiguous strides was found to be $N=4$, which means that the front-view Kinect records useful skeleton tracking data for about 3 m. However, also trial walks with 5 and 3 strides were found as this number depends also on the speed and the leg length of the subject.

### Validation

**Fig. 11:**
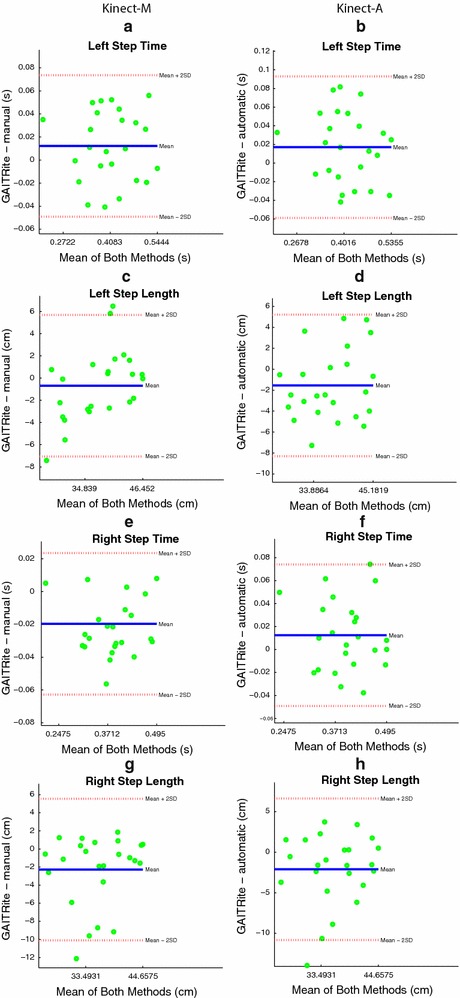
Bland and Altman plots. On the *left side*: **a**, **c**, **e**, **g** show the comparison between GAITRite (criterion) and Kinect-M, where gait parameters are estimated with heel strike and toe-off instants computed manually. On the *right side*: **b**, **d**, **f**, **h** show the comparison between GAITRite (criterion) and Kinect-A, where gait parameters are computed fully automatically. *Solid lines* indicate the mean difference between criterion and the system to be validated. *Dashed lines* indicate the limits of agreement ($\pm 1.96$ SD). The parameters compared are left step time, left step length, right step time, and right step length

**Fig. 12:**
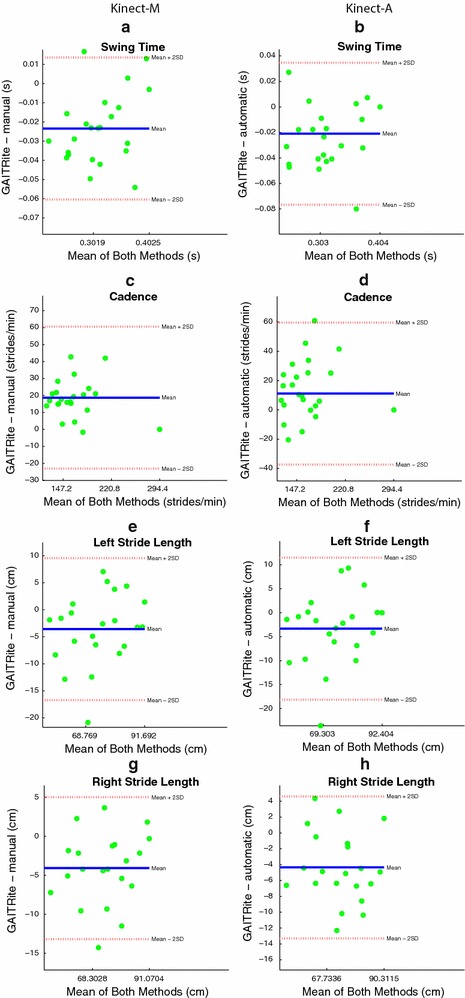
Bland and Altman plots. On the *left side*: **a**, **c**, **e**, **g** show the comparison between GAITRite (criterion) and Kinect-M, where gait parameters are estimated with heel strike and toe-off instants computed manually. On the *right side*: **b**, **d**, **f**, **h** show the comparison between GAITRite (criterion) and Kinect-A, where gait parameters are computed fully automatically. *Solid lines* indicate the mean difference between criterion and the system to be validated. *Dashed lines* indicate the limits of agreement ($\pm 1.96$ SD). The parameters compared are swing time, cadence, left stride length, and right stride length

**Fig. 13:**
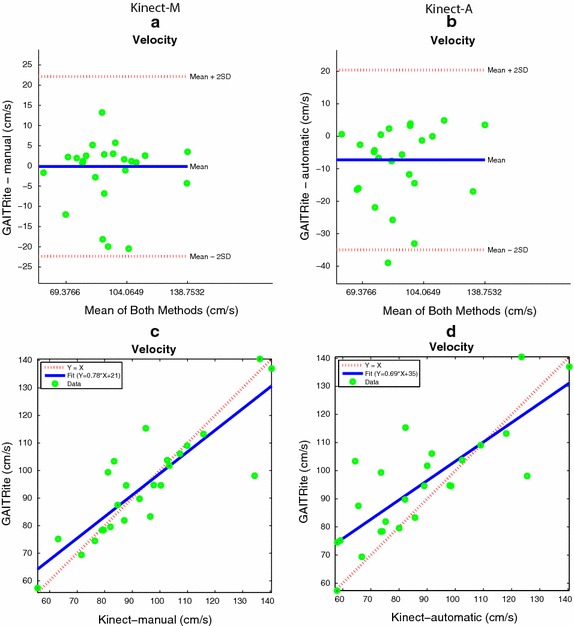
Bland and Altman plots and Scatter plots for the Velocity. On the *left side*: **a**, **c** show the comparison between GAITRite (criterion) and Kinect-M, where the velocity is estimated with heel strike and toe-off instants computed manually. On the *right side*: **b**, **d** show the comparison between GAITRite (criterion) and Kinect-A, where the velocity is computed fully automatically. *Solid lines* indicate the mean difference between criterion and the system to be validated for the Bland and Altman plots, as well as the linear best-fit for the scatter plot. *Dashed lines* indicate the limits of agreement ($\pm 1.96$ SD) for the Bland and Altman plot, as well as the identity line for the scatter plot

**Fig. 14:**
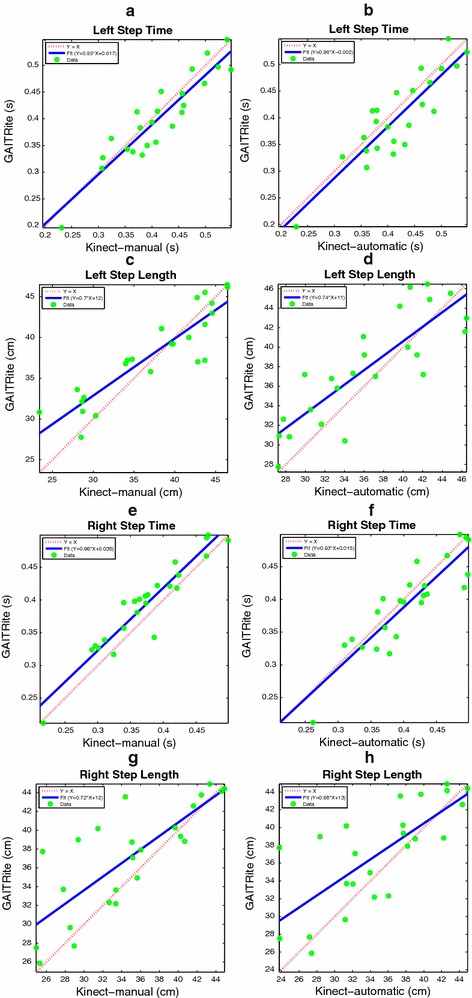
Scatter plots. On the *left side*: **a**, **c**, **e**, **g** show the comparison between GAITRite (criterion) and Kinect-M, where gait parameters are estimated with heel strike and toe-off instants computed manually. On the *right side*: **b**, **d**, **f**, **h** show the comparison between GAITRite (criterion) and Kinect-A, where gait parameters are computed fully automatically. *Solid lines* indicate the linear best-fit. *Dashed lines* indicate the identity line. The parameters compared are left step time, left step length, right step time, and right step length

**Fig. 15:**
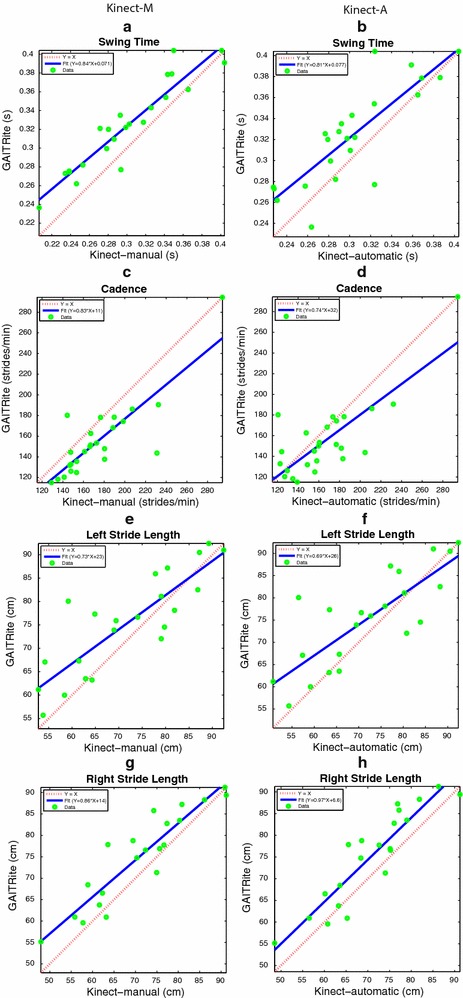
Scatter plots. On the *left side*: **a**, **c**, **e**, **g** show the comparison between GAITRite (criterion) and Kinect-M, where gait parameters are estimated with heel strike and toe-off instants computed manually. On the *right side*: **b**, **d**, **f**, **h** show the comparison between GAITRite (criterion) and Kinect-A, where gait parameters are computed fully automatically. *Solid lines* indicate the linear best-fit. *Dashed lines* indicate the identity line. The parameters compared are swing time, cadence, left stride length, and right stride length

The level of agreement between the manual estimation approach (Kinect-M) and the GAITRite, and between Kinect-A and the GAITRite is evaluated. The gait parameters under consideration are the left and right step time, the cadence, the average swing time, the left and right stride length, the left and right step length , and the velocity. Figures  11, 12 and 13a, b show the Bland and Altman plots, where for each gait parameter the Kinect-M plot and the Kinect-A plot are next to each other to facilitate their visual comparison. All data were normally distributed (*p*-value $<0.0002$, and $<0.0015$ for Kinect-M right step length), and exhibited a mean bias but no heteroscedasticity and no proportional error. Bland–Altman bias and limits of agreement (LoA) are reported in Table 2 for Kinect-M, and Table 3 for Kinect-A. Figs. 13c, d, 14 and 15, instead, show the scatter plots, where, again, for each gait parameter the Kinect-M plot and the Kinect-A plot are next to each other to facilitate their visual comparison. Table 4 reports the means and standard deviations (SDs) of the parameters for the three methods.

**Table 2:** Agreement and repeatability—Kinect-M

Gait parameters	$\rho$	CCC (95 % CI)	ICC (95 % CI)	Bias (95 % LoA)	PE (%)	Repeatability	
						Absolute	Mean %
Left step time (s)	0.926	0.91 (0.81–0.96)	0.95 (0.89–0.98)	0.012 (−0.048 to 0.072)	15.01	0.050	12.19
Right step time (s)	0.948	0.90 (0.81–0.95)	0.95 (0.88–0.98)	−0.020 (−0.062 to 0.023)	11.29	0.071	18.91
Cadence (strides/min)	0.838	0.83 (0.66–0.92)	0.83 (0.65–0.92)	−1.230 (−21.259 to 18.798)	25.83	20.75	13.44
Swing time (s)	0.939	0.83 (0.69–0.91)	0.91 (0.80–0.95)	−0.023 (−0.060 to 0.013)	11.92	0.050	16.66
Left stride length (cm)	0.848	0.79 (0.59–0.90)	0.89 (0.75–0.95)	−3.583 (−16.447 to 9.282)	17.85	10.208	14.23
Right stride length (cm)	0.920	0.85 (0.70–0.93)	0.92 (0.80–0.95)	−4.085 (−13.009 to 4.840)	12.46	10.107	14.23
Left step length (cm)	0.900	0.86 (0.74–0.93)	0.93 (0.82–0.96)	−0.681 (−6.927 to 5.565)	16.94	4.227	11.34
Right step length (cm)	0.792	0.73 (0.50–0.86)	0.92 (0.82–0.96)	−2.281 (−9.947 to 5.385)	21.76	4.858	13.96
Velocity (cm/s)	0.855	0.84 (0.68–0.92)	0.85 (0.69–0.93)	−3.036 (−13.904 to 7.832)	23.33	12.796	13.96

Pearson’s correlation ($\rho$), CCC, ICC, Bland-Altman bias and LoA’s, percentage error (PE) (computed as $100 \times (4 \text { SD of bias})/ (\text {Mean}_{\text {Kinect-M}} + \text {Mean}_{\text {GAITRite}})$), and repeatability coefficients for the manual estimation method (Kinect-M)

**Table 3:** Agreement and repeatability—Kinect-A

Gait parameters	$\rho$	CCC (9 5% CI)	ICC (95 % CI)	Bias (95 % LoA)	PE (%)	Repeatability	
						Absolute	Mean %
Left step time (s)	0.881	0.85 (0.70–0.93)	0.92 (0.81–0.95)	0.017 (−0.057 to 0.092)	18.52	0.061	14.63
Right step time (s)	0.892	0.87 (0.73–0.94)	0.93 (0.84–0.98)	0.012 (−0.048 to 0.073)	15.43	0.071	17.50
Cadence (strides/min)	0.796	0.79 (0.58–0.90)	0.79 (0.59–0.90)	2.368 (−21.104 to 25.842)	29.92	23.380	14.79
Swing time (s)	0.833	0.75 (0.53–0.88)	0.86 (0.71–0.95)	−0.021 (−0.075 to 0.033)	17.58	0.061	20.00
Left stride length (cm)	0.805	0.76 (0.53–0.88)	0.87 (0.76–0.93)	−3.326 (−17.846 to 11.194)	20.11	10.230	14.21
Right stride length (cm)	0.912	0.83 (0.66–0.92)	0.91 (0.72–0.94)	−4.346 (−13.119 to 4.427)	12.27	10.099	14.27
Left step length (cm)	0.840	0.80 (0.61–0.90)	0.89 (0.73–0.95)	−1.544 (−8.167 to 5.079)	18.17	4.198	11.53
Right step length (cm)	0.737	0.69 (0.42–0.84)	0.82 (0.65–0.92)	−2.108 (−10.656 to 6.441)	24.21	5.127	14.66
Velocity (cm/s)	0.793	0.77 (0.57–0.88)	0.77 (0.55–0.89)	−1.276 (−15.966 to 13.413)	31.24	13.674	14.64

Pearson’s correlation ($\rho$), CCC, ICC, Bland-Altman bias and LoA’s, percentage error (PE) (computed as $100 \times (4 \text { SD of bias})/ (\text {Mean}_{\text {Kinect-A}} + \text {Mean}_{\text {GAITRite}})$), and repeatability coefficients for the automatic estimation method (Kinect-A)

**Table 4:** Gait parameter statistics

Gait parameters	Kinect-M		Kinect-A		GAITRite	
Left step time (s)	0.414 (0.080)		0.419 (0.073)		0.402 (0.080)	
Right step time (s)	0.374 (0.067)		0.406 (0.064)		0.394 (0.067)	
Cadence (strides/min)	154.45 (32.789)		158.48 (37.092)		157.068 (36.248)	
Swing time (s)	0.301 (0.053)		0.303 (0.049)		0.324 (0.048)	
Left stride length (cm)	71.736 (12.369)		71.993 (12.428)		75.319 (10.672)	
Right stride length (cm)	71.029 (11.639)		70.768 (10.241)		75.114 (10.873)	
Left step length (cm)	37.274 (7.042)		36.411 (6.218)		37.955 (5.482)	
Right step length (cm)	34.799 (6.292)		34.972 (6.243)		37.080 (5.717)	
Velocity (cm/s)	91.632 (20.69)		93.391 (24.141)		94.665 (19.571)	

Mean (SD) values for the manual estimation method (Kinect-M), the automatic estimation method (Kinect-A), and the GAITRite

Tables 2 and 3 report additional agreement parameters for Kinect-M and Kinect-A, respectively. Levels of agreement are considered to be excellent, good, moderate, or modest if $\rho$, CCC, or ICC are greater than 0.9, 0.8, 0.7, or 0.5, respectively. For Kinect-M, most parameters show excellent relative agreement ($\rho >0.9$), and good to excellent overall agreement (CCC $>0.8$), with mostly excellent absolute agreement (ICC $>0.9$). For Kinect-A the relative agreement is mostly good and excellent ($\rho >0.8$), with moderate and good overall agreement (CCC $>0.7$), and with good and excellent absolute agreement (ICC $>0.8$).

The repeatability test shows that with probability greater than 95 %, the measurement of a parameter will differ from the previously measured value by an amount less than those reported in Tables 2 and 3. For Kinect-M in particular, the repeatability is very good for most of the parameters (<15 % of the mean), and good (<20 % of the mean) for the right step time, and the swing time. The same behavior is observed for Kinect-A.

## Discussion

Table 1 confirms the importance of the design choices made to address the challenge of performing an accurate segmentation in presence of a very high variability of the temporal trajectories of skeleton vectors in children. In particular, $US_L$ shows the poorest performance because it only models uniform scaling. $SDTW_L$ adds to $US_L$ the ability to account for non-uniform scaling, and leads to an improvement. $CSDTW_L$, instead, forces the strides to be contiguous, further improving the performance. The first step of Kinect-A improves results even more because uniform and non-uniform scaling are handled jointly by $SSWM_L$, not separately ($US_L$ followed by $SDTW_L$). Finally, the second step of Kinect-A (last row of Table 1), refines the segmentation by imposing contiguous strides. Note that $SSWM_L$ outperforms not only the two-steps $US_L\!-\!SDTW_L$, but also their contiguous refinement (fourth row of Table 1). Overall, the accuracy of Kinect-A is excellent (AoD and Rand index ${>}0.9$), and the precision of the instant estimates is good (i.e., around or less than 20 % of the means in Table 4, 95 % of the time).

Kinect-A is also computationally efficient. Indeed, with a Matlab implementation on a low-end PC, the running time of $SSWM_L$ applied to a trial walk with length $n=135$ is 4.11 s, and the running time of $CSDTW_L$ is 7.05 s. On the other hand, $SWM_L$ takes 75 min even when the length of the matching subsequence is constrained in the range $[\lfloor 0.8~m \rfloor , \lceil 1.2m \rceil ]$, and the template has length *m*. Therefore, $SSWM_L$ provides a remarkable 1000 speedup factor, which is essential for implementing Kinect-A in a low-cost platform with limited computing power.

Kinect-M represents an upper bound on the agreement, and Kinect-A approaches it with an average percentage deterioration of 5.5 % for the relative agreement, of 6.1 % for the overall agreement, and of 4.5 % for the absolute agreement. The Bland-Altman bias, instead, on average changes only by 2.18 % of the mean of the corresponding GAITRite parameter. In terms of PE, there is an average deterioration of 2.76 percentage points. Overall, this means that Kinect-A can reach levels of agreement very close to those achievable by a manual inspection of Kinect data, which is extremely encouraging. The temporal parameters are those that exhibit more deterioration, especially the swing time. This is probably due to the limit imposed by the temporal resolution of the skeleton tracking, which is 30 frames per second.

Kinect-A repeatability on average deteriorates only by 0.71 points, compared to Kinect-M, which is remarkable. In particular, it remains very good even when the agreement with the GAITRite decreases a bit more, like for the right step length. For temporal parameters the repeatability worsens on average by 1.2 points, and by only 0.23 points for spatial parameters. This highlights that temporal resolution affects repeatability, as is also suggested by comparing the repeatability of cadence and swing time. The former is better because less sensitive to the resolution, since it is related to measuring time intervals much larger than those measured for the swing time. Finally, we note that very good repeatability parameters, as often observed in both Kinect-M and Kinect-A, are also indicative of the fact that differences between trial walks of the same subject are limited.

Agreement and repeatability are affected by temporal resolution and skeleton tracking quality. However, while temporal resolution appears to have a stronger impact on the Kinect-A performance with respect to Kinect-M, this is not the case for the agreement with the GAITRite in general. Indeed, spatial parameters have worse levels of agreement than temporal parameters; highlighting that tracking quality, rather than temporal resolution, should be responsible for this difference.

## Limitations and future work

This section describes the major limitations of the proposed approach, which might suggest future directions of investigation. An importan aspect that has not been fully studied is the effect of various sources of noise onto the gait parameters estimation. The Kinect skeleton tracking data is affected by noise in the spatial and temporal domain. In this work we acquired data with the default joint filtering option of the SDK turned on to filter out small jitters and maintain a very low latency. This allows smoothing the spatial noise across different frames to minimize jittering and stabilize the joint positions over time. In addition, the temporal sampling of the Kinect was assumed to be deterministic, with a frequency of 30 Hz. However, the sampling has a Gaussian jitter, as reported also in [75, 76]. For example, [75] reports a sampling period with mean 33.4 ms, and SD 3.7 ms.

Although a full investigation of the temporal jittering effects should be addressed in future research, a very simplified analysis allows gauging to what extent jittering affects our approach. For example, if we are measuring a stride time of 0.8 s (essentially the average stride time of our population), we expect to sample the stride 24 times. Therefore, by assuming the sequence of sampling periods to be made by independent and identically distributed Gaussian variables, the stride time becomes a Gaussian variable with mean $24 \times 33.4$ ms, and SD $\sqrt{24} \times 3.7$ ms. However, according to (12), the average stride time *R* is computed over 3 trials, each of which has an average of 4 strides. Therefore, *R* is a Gaussian variable with mean $24 \times 33.4$ ms, and SD $\sqrt{24} \times 3.7 / \sqrt{3 \times 4} = \sqrt{2} \times 3.7$ ms. This means that *R* has a coefficient of variation due to the temporal jitter of 0.65 %, which is small, suggesting that a fixed sampling frequency of 30 Hz is a plausible working assumption, as confirmed by the promising validation results.

The Kinect skeleton tracking data is also affected by the distance between the Kinect and the individual. The further away is the individual, and the lower is the tracking accuracy. Therefore, single cycle step lengths or step times will be affected by greater errors if they correspond to step cycles at the beginning of the trial walk, which is further away, whereas if they correspond to later steps, they will provide more accurate quantities. However, since gait parameters are computed by averaging over several step cycles, this has the effect of leveling off a lot of the effects induced by the dependency upon the distance of the accuracy. While this might sound reasonable and intuitive, a thorough investigation of this dependency should be addressed in future work.

Another issue left unexplored is the effect of stratification. The stride template models are learned with data from the entire children age range (2–4 years). Therefore, as long as the child being tested has an age within that range, Kinect-A is expected to work. While this is a strength of the approach, it would still be possible to learn different stride template models for different age ranges, or for different children leg length ranges. In this way, a more specific template model could be preselected based on the child age, or could even be automatically selected, based on the automatic estimation of the leg length from the Kinect skeleton tracking data. A future investigation should establish whether using stratified template models will significantly increase the accuracy and precision of the approach.

Although the Kinect has had a powerful impact on several clinical applications [45, 46], updated technology might further expand it, even for gait analysis applications. It is expected that improvements in the temporal resolution and in the quality of the skeleton tracking, coming with the updated versions of Kinect [28], will produce better concurrent validity and repeatability. Determining the size of such improvements, and to what extent Kinect-A can be used to replicate the large set of parameters computed by the GAITRite, will be the subject of future research.

An important future direction for expanding the horizon of Kinect-A is its application to an adult population. In principle, this could be done as long as stride template models are learned for this specific case. However, the size of adults leads to proportional stride lengths increases, and to a reduced amount of strides captured by the system during a single trial walk. Therefore, this aspect as well as the different probability distribution of the skeleton tracking information, will have a nontrivial effect on the gait parameters that will need to be investigated.

Finally, we stress the fact that this study has introduced Kinect-A for children’s gait analysis, but the validation has been limited to healthy subjects. Therefore, perhaps the most relevant extension of Kinect-A should be operated with the goal in mind of doing children’s gait analysis on any subject, regardless of her health status.

## Conclusions

This work has proposed the Kinect-A method for the automated estimation of children’s gait parameters, based on the Microsoft Kinect, and has assessed its concurrent validity against the GAITRite on healthy subjects. The core of Kinect-A is based on bringing together maximum likelihood estimation, uniform and non-uniform scaling estimation, and subsequence matching principles. This approach has demonstrated the ability to cope with the high variability of healthy children’s skeleton tracking data acquired by the Kinect by providing excellent temporal segmentation accuracy, and good precision ,computed against the ground-truth obtained with the specialized manual annotation procedure of Kinect-M. Moreover, the approach is computationally efficient, with low computing power needs.

A study conducted with healthy children has shown that Kinect-A has good concurrent validity against the GAITRite, as well as very good repeatability. In particular, on a range of 9 gait parameters, the relative and absolute agreements were found to be good and excellent, and the overall agreements were found to be good and moderate. Moreover, we found that the agreement and repeatability parameters of Kinect-A very closely approached those of Kinect-M, which represents an upper bound. In particular, the agreement is found to have an average percentage deterioration of $5.37~\%$, and the repeatability is found to deteriorate by 0.71 points on average. Despite the limited evaluation conditions based on healthy subjects, the results obtained with Kinect-A represent a step forward in that they encourage further development, with the goal of deploying a fully functional low-cost, parent-operable, portable system for in-home monitoring of gait in children (age 2–4 years), which can operate in actual rehabilitation intervention scenarios.
